# Bowel preparation for colonoscopy in children: a survey of the Italian society of pediatric gastroenterology, hepatology and nutrition

**DOI:** 10.1186/s13052-026-02227-4

**Published:** 2026-03-19

**Authors:** Elvira Difrancisca, Antonio Corsello, Martina Genduso, Luca Maria Antoniello, Mara Cananzi, Sara Renzo, Paola De Angelis, Francesca Cavataio, Claudio Romano

**Affiliations:** 1 Pediatric Gastroenterology Unit, Children’s Hospital “G. Di Cristina”, ARNAS Civico-Di Cristina-Benfratelli, Palermo, Italy; 2 Department of Clinical-Surgical, Diagnostic and Pediatric Sciences, University of Pavia, Pavia, Italy; 3 School of Pediatrics, University Department PROMISE “G. D’Alessandro”, University of Palermo, Palermo, Italy; 4 Pediatric Surgery, University Hospital of Padua, Padua, Italy; 5 Unit of Pediatric Gastroenterology, Digestive Endoscopy, Hepatology and Care of the Child with Liver Transplantation, Department for women’s and Children’s Health, University Hospital of Padua, Padua, Italy; 6 Gastroenterology and Nutrition Unit, Meyer Children’s Hospital IRCCS, Florence, Italy; 7 Gastroenterology and Nutrition Unit, Bambino Gesù Children Hospital IRCCS, Rome, Italy; 8 Pediatric Gastroenterology and Cystic Fibrosis Unit, Department of Human Pathology In Adulthood and Childhood, University of Messina, Messina, Italy

**Keywords:** Pediatric endoscopy, Colonoscopy, Bowel preparation, Polyethylene glycol, PEG, Sodium picosulfate, Guidelines

## Abstract

**Background:**

Adequate bowel preparation is critical for the success of pediatric colonoscopy. International guidelines recommend standardized low-volume regimens, although several studies also support individualized, patient-tailored approaches. Despite this, real-world pediatric practices remain highly heterogeneous. This national survey aimed to investigate current bowel preparation practices for pediatric colonoscopy in Italy and compare them with international recommendations.

**Methods:**

A nationwide electronic survey was conducted among centers affiliated with the Italian Society of Pediatric Gastroenterology, Hepatology and Nutrition (SIGENP). The questionnaire explored bowel preparation protocols, adherence to international guidelines, and the occurrence of adverse events.

**Results:**

A total of 39 centers participated in the survey. Sodium picosulfate and polyethylene glycol with electrolytes were the most commonly used preparation agents, although high-volume solutions are often poorly tolerated. Only 43.6% of centers adhered to international guidelines recommending low-volume regimens, whereas 12 centers (30.8%) adopted multiple preparation strategies tailored to clinical indications and patient-specific characteristics. Preparation setting (home vs. hospital), agent selection, and use of enemas or laxatives varied significantly. Adverse events were uncommon, and most centers considered bowel preparation stressful for children. The Boston Bowel Preparation Scale was the most commonly used tool for assessing bowel cleanliness. Nearly all clinicians expressed a strong need for national pediatric guidelines.

**Conclusions:**

This survey, conducted by SIGENP, reveals substantial variability in bowel preparation practices for pediatric colonoscopy across Italian centers, reflecting a trend also observed in other countries. Our results underscore the urgency of developing and implementing pediatric bowel preparation guidelines to ensure safety, improve tolerability, and reduce heterogeneity of practice. Further research is needed to assess whether such variability may offer benefits through individualized approaches tailored to the specific needs of each child.

**Supplementary Information:**

The online version contains supplementary material available at 10.1186/s13052-026-02227-4.

## Background

Bowel preparation is a critical prerequisite for achieving adequate visualization of the intestinal lumen during a colonoscopy, enabling the detection of mucosal lesions and other abnormalities. However, it is estimated that more than 25% of pediatric patients undergo a colonoscopy with insufficient bowel cleansing, which may result in missed diagnoses, reduced procedural safety, prolonged procedure time, repeat procedures, and need for additional anesthesia [1, 2]. These complications not only affect patient outcomes but also contribute to increased healthcare costs. An optimal bowel preparation should be safe, palatable, and compatible with the patient’s everyday routine.

Commonly used agents include polyethylene glycol (PEG)-3350 solutions, PEG with electrolytes (PEG-ELS), and sodium picosulfate, with evidence suggesting comparable efficacy among these options [3, 4]. Pediatric studies in line with adult literature have demonstrated that low-volume preparation regimens — such as sodium picosulfate/magnesium citrate, PEG/ascorbate and PEG with bisacodyl, typically administered as 2 L in one or two doses — are noninferior to high-volume PEG regimens (PEG at a dose of 100 mL/kg, up to a maximum of 4 L) in terms of bowel cleansing efficacy. Importantly, low-volume regimens are better tolerated by pediatric patients and associated with a lower incidence of nasogastric tube placement [5]. The European Society of Gastrointestinal Endoscopy (ESGE) guidelines recommend a low-fiber diet on the day before colonoscopy, followed by a split-dose regimen with preparations, preferably low-volume, based on PEG or sodium picosulfate [6]. The European Society for Paediatric Gastroenterology, Hepatology and Nutrition (ESPGHAN) guidelines also strongly recommend low-volume preparations for bowel cleansing in children. Preferred options, supported by high-quality evidence, include PEG with ascorbate or sodium picosulfate combined with magnesium citrate [6, 7].

Despite these recommendations, no universally accepted pediatric protocol exists, and real-world practices vary considerably among institutions [2, 6, 7]. While guideline-based standardization is fundamental to ensure safety, efficacy and comparability of clinical outcomes, the heterogeneity of pediatric populations often requires patient-centered personalization, considering age, comorbidities, tolerability, previous experiences, and family support.

While international guidelines provide valuable recommendations, the Italian healthcare context presents unique challenges that warrant consideration of region-specific guidance. The national availability of bowel preparation agents may differ from that of the other European countries, with limited access to low-volume formulations approved for pediatric use and variable reimbursement policies across regions. Additionally, the regulatory framework governing off-label use of medications in Italy could present regional variability in pharmaceutical procurement and further restrict access to recommended preparations. These factors, combined with organizational differences in pediatric gastroenterology services across the country, justify the development of guidelines that account for the local healthcare system while maintaining adherence to evidence-based principles.

In this context, a combined approach that aligns guideline-driven protocols with patient-tailored adjustments is likely to yield the highest effectiveness in bowel preparation. In this study, we investigated real-life practices for bowel preparation in pediatric colonoscopy across Italy and compared them with the international recommendations of ESGE and ESPGHAN. Furthermore, this study aims to identify barriers to guideline adherence and gather clinician perspectives on the potential implementation of standardized national protocols.

## Methods

To evaluate current practices for bowel preparation before colonoscopy across Italian pediatric hospitals, a nationwide multiple-choice electronic survey was distributed to centers (general pediatric units, pediatric gastroenterology units, pediatric surgery units) affiliated with the Italian Society of Pediatric Gastroenterology, Hepatology and Nutrition (SIGENP).

The survey consisted of 31 questions designed to explore different aspects of current practice related to pediatric bowel preparation for colonoscopy. The questionnaire covered 12 key domains, including operator and center characteristics, procedural settings, bowel preparation regimens, adherence to international guidelines, tolerability, and clinicians’ perspectives on the need for national recommendations (Table 1).

**Table 1:** Main domains of the national survey on bowel preparation for pediatric colonoscopy

Domain	Key Topic
Center and operator characteristics	Type of department and specialists performing pediatric colonoscopy
Endoscopy setting	Procedure frequency and environment
Bowel preparation: setting, criteria, regimen, documentation	Where and how preparation is performed; Agents and combinations used; Use of quality scoring and reporting
Guidelines and need for national guidelines	Adherence to international recommendations (ESGE, ESPGHAN, NASPGHAN), clinical perspectives
Hospital-provided formulation and adjunctive measures	Use of hospital-provided products, protocols, diet etc.
Colonoscopy	Dose regimen and timing
Tolerability and complications	Issues regarding Adverse Event and tolerability
Supportive measures	Use of antiemetics and/or nasogastric tubes

Specifically, the survey investigated:

- who performs pediatric endoscopies, the settings in which these procedures are conducted, and the monthly frequency of colonoscopies;
- the criteria used to determine whether bowel preparation is conducted in the hospital or at home;
- the most commonly adopted regimens and their potential complications or adverse events;
- adherence to ESGE/ESPGHAN guidelines, including dietary recommendations and the use of adjunctive measures such as enemas or laxatives;
- timing and dosing strategies (single-dose vs. split-dose regimens);
- methods used to assess and document bowel cleanliness.

The full questionnaire is available in the Supplementary Material (Supplementary Table 1).

The survey, published on the SIGENP website, was accessible from 1 June to 31 July 2024. Responses were collected in a Microsoft Excel database. Descriptive statistics were used to analyze the survey responses. Categorical variables are presented as frequencies and percentages. Data were processed using R statistical software. Ethical approval and consent were not required due to the study design and local regulations, and all methods were performed following the ethical standards of the Declaration of Helsinki. Participation was voluntary and anonymized.

## Results

A total of 39 centers across Italy participated in the survey. Most were located in Northern Italy (*n* = 22; 56.4%), followed by Southern Italy and the Islands (*n* = 8; 20.5%) and Central Italy (*n* = 9; 23.1%). The highest number of responses came from Rome (*n* = 4), Milan (*n* = 3), Naples (*n* = 3), and Alessandria (*n* = 3) (Supplementary Table 2).

All respondents were pediatric specialists working in different clinical settings: 20 (51.3%) in general pediatric units, 15 (38.5%) in pediatric gastroenterology units, and 4 (10.2%) in pediatric surgery units. Endoscopies were performed by adult gastroenterologists in 15 centers (38.5%), by pediatric gastroenterologists in 11 centers (28.2%), and by pediatric surgeons in 2 centers (5.1%); mixed teams operated in 11 centers (28.2%). Procedures were carried out in both endoscopy units and operating rooms with comparable distribution. The number of monthly colonoscopies varied widely: 13 centers (33.3%) performed fewer than 5 procedures, 13 (33.3%) performed 5–10, 7 (17.9%) performed 10–20, and 6 (15.4%) performed more than 20 (Table 2).

**Table 2:** – Characteristics of unit, specialists and number of procedures (*n* = 39 centers)

**Type of unit**	**No. of centers (%)**
General pediatric unit	20 (51.3%)
Pediatric gastroenterology unit	15 (38.5%)
Pediatric surgery unit	4 (10.2%)
**Specialist performing procedures**	**No. of centers (%)**
Adult gastroenterologist	15 (38.5%)
Pediatric gastroenterologist	11 (28.2%)
Pediatric surgeons	2 (5.1%)
Mixed Teams	11 (28.2%)
**Monthly number of colonoscopies**	**No. of centers (%)**
5 or less	13 (33.3%)
6 - 10	13 (33.3%)
> 10 and < = 20	7 (17.9%)
20 or more	6 (15.4%)

In 27 centers (69.2%), the decision to perform bowel preparation at home or in hospital was based on predefined clinical criteria, most commonly patient age (24/27), family and patient compliance (22/27), and comorbidities (15/27), particularly neurological or metabolic disorders. Additional factors included the child’s clinical condition at the time of the procedure (4/27), whether the colonoscopy was a first or follow-up examination (3/27), geographical distance from the endoscopy center (3/27), and a history of inadequate bowel cleansing (2/27). Nine centers (23.1%) performed bowel preparation exclusively in hospital, whereas three centers (7.7%) always performed it at home.

Sodium picosulfate and polyethylene glycol with electrolytes (PEG-ELS) were the most frequently used bowel preparation agents, adopted by 30/39 (76.9%) and 25/39 (64.1%) centers, respectively (Table 3). Sodium picosulfate was used as a single regimen in 13 centers (33.3%), whereas PEG-ELS alone was used in 9 centers (23.1%), including 5 using high-volume and 4 using low-volume regimens. PEG 3350 without electrolytes was not used as a single preparation in any center. Several centers adopted more than one regimen, tailoring the approach based on clinical indication, tolerability, or patient characteristics. Specifically, 6 centers (15.4%) alternated between sodium picosulfate and high-volume PEG-ELS, 3 (7.7%) between sodium picosulfate and low-volume PEG-ELS, 5 (12.8%) offered three options (sodium picosulfate, high-volume PEG-ELS, and low-volume PEG-ELS), 2 (5.1%) used sodium picosulfate and PEG 3350, and 1 center (2.6%) reported using all four regimens (Table 3).

**Table 3:** Distribution of bowel preparation regimes and type of preparations (*n* = 39)

Preparation regimes	Description	No. of centers (%)
Sodium picosulfate alone	Used as the sole bowel preparation agent	13 (33.3%)
PEG – ELS alone	Used exclusively; includes both high and low-volume regimens	9 (23.1%)
Sodium picosulfate + High-Volume PEG-ELS	Alternative regimens depending on patients characteristics	6 (15.4%)
High-Volume PEG-ELS	PEG – ELS 4 L or 100 mL/kg up to 4 L	5 (12.8%)
Sodium picosulfate + High and Low-Volume PEG-ELS	Three different options tailored to the patient	5 (12.8%)
Low-Volume PEG-ELS	PEG – ELS 2 L or reduced-dose regimen	4 (10.3%)
Sodium picosulfate + Low-Volume PEG-ELS	Alternative regimens according to clinical indications	3 (7.7%)
Sodium picosulfate + PEG 3350	PEG without electrolytes	2 (5.1%)
All four regimen available	Sodium picosulfate + High- and Low-Volume PEG-ELS, PEG 3350	1 (2.6%)
**Type**	**Number of centers (%)**	
Single	22 (56.4%)	
Combination	17 (43.6%)	

Seventeen centers (43.6%) reported adherence to ESGE/ESPGHAN recommendations, which suggest the use of low-volume preparations. The remaining 22 centers (56.4%) either diverged from the recommended agents or employed high-volume regimens.

Most centers (33/39) used hospital-provided bowel preparations; however, 24 of these reported dissatisfaction due to poor palatability and inappropriate volumes for pediatric use, and 9 centers limited their use to selected clinical circumstances (e.g., allergy, age restrictions, clinical severity, or compliance issues). Six centers did not use hospital-provided preparations due to the same concerns and because these products were not recommended for pediatric use. Additionally, five centers reported switching to an alternative regimen following inadequate cleansing.

High-volume PEG-ELS preparations were used in 21 centers. According to the product information, they are contraindicated in children younger than 8 years or weighing less than 20 kg; of the 21 centers using them, 10 reported using them in line with this restriction, while 11 did so regardless of age or weight. Low-volume PEG-based formulations (e.g., Plenvu, Clemsia, Moviprep), which are contraindicated in patients under 18 years of age, were regularly used in 9 centers. Sodium picosulfate was used in 31 centers (79.5%); despite its contraindication in active inflammatory bowel disease, 23 centers reported routine use in this population.

Osmotic laxatives were prescribed in 29 centers, although only two reported routine use. They were mainly administered to manage constipation (21/29) or following inadequate bowel cleansing (14/29), typically starting 2–3 days prior to the procedure. Ten centers did not prescribe osmotic laxatives. A low-fiber diet was recommended by 69% of centers, whereas rectal enemas were used only in cases of insufficient cleansing in 27 centers. Patients were generally instructed to complete bowel preparation within 4–6 h in 22 centers, while 15 centers followed the manufacturer’s instructions.

Colonoscopies were predominantly scheduled in the morning (26/39), while 11 centers arranged timing based on endoscopy suite availability and only two performed procedures exclusively in the afternoon. For afternoon procedures, split-dose regimens were most commonly used (11 centers). Five centers postponed the start of preparation to as late as possible on the day before the procedure; one adopted same-day preparation, one included an evacuative enema, and one strictly followed the manufacturer’s leaflet. Nine centers did not modify the standard regimen for afternoon procedures.

Most centers used a nasogastric tube only when oral intake was not possible. One center routinely used it in children under 10 years of age, whereas none used it routinely in children under 5. Poor taste and difficulty ingesting large volumes were reported as the most relevant tolerability problems (28 centers; 71.7%). Nausea and vomiting were reported in 18 centers (46.2%), abdominal cramps in two centers, and 21 centers reported at least one of these symptoms. Ondansetron was considered the antiemetic of choice by centers using antiemetics (96%), although it was administered in only 18 centers, whereas 21 did not use antiemetics. Adverse events were infrequent. Hypotension was reported in five centers, whereas most centers (34/39) reported no adverse events. Nearly all centers (38/39) considered bowel preparation a stressful procedure for children.

Bowel preparation was documented in all participating centers. The Boston Bowel Preparation Scale (BBPS) was used in 26 centers (68.4%), while seven centers relied exclusively on qualitative descriptors (e.g., “adequate,” “insufficient”), and five reported using both approaches. Written and oral instructions were provided in 49% of centers, 36% provided written material only, and 15% relied solely on oral communication. Finally, nearly all centers (37/39; 94.8%) expressed the need for national Italian guidelines to standardize pediatric bowel preparation protocols for pediatric colonoscopy.

## Discussion

This survey achieved good national coverage across Italy, and no significant geographical differences were observed in bowel preparation practices. The participating centers included both general pediatric units and pediatric gastroenterology units in comparable proportions, offering a representative overview of current national practice. Pediatric endoscopic procedures are still frequently performed by adult gastroenterologists, mainly due to their extensive procedural experience and higher endoscopic workload. Indeed, only a minority of centers performed more than 20 pediatric colonoscopies per month, suggesting that limited case volumes may restrict the opportunity for continuous pediatric-focused procedural training. Nevertheless, an increasing involvement of pediatric gastroenterologists with specific training was observed, indicating a gradual shift toward more pediatric-centered endoscopy services [1, 2].

The choice between home-based and hospital-based bowel preparation varies considerably across centers. Most centers adopt a flexible approach, tailoring the regimen to the needs of the child and family, while others prefer hospital-based preparation to ensure closer monitoring; a minority, instead, have reported positive experiences with home-based protocols.

Sodium picosulfate was the most frequently used bowel preparation agent in Italian centers, followed by PEG-ELS (30/39 vs. 25/39 centers), consistent with evidence confirming its efficacy and favorable safety profile in pediatric patients [5, 8, 9]. High-volume PEG-ELS remains widely adopted due to its availability in hospital settings and well-established safety record; nonetheless, it is often associated with poor palatability, the need for large fluid intake and gastrointestinal discomfort, which may require nasogastric tube administration or the use of antiemetics. Low-volume PEG-ELS regimens, also recommended by the scientific societies, are increasingly used, although currently adopted in a smaller proportion of centers (9/39). The widespread off-label use of different preparations across various age groups and clinical scenarios further suggests a perceived acceptable level of safety in daily clinical practice. Reported adverse events were infrequent, reinforcing this perception; nonetheless, careful clinical monitoring throughout the entire process remains essential.

A relevant finding of our survey is that several centers (12/39) did not rely on a single standardized protocol but instead adopted multiple strategies based on clinical needs, patient characteristics, tolerability, or previous experiences. This individualized approach may represent a strength in pediatric practice, where personalization is often necessary due to substantial clinical variability. However, such variability also contributes to notable heterogeneity in clinical practice and may hinder the development of shared and uniform care pathways.

At the same time, only 43.6% of centers adhered to the ESGE/ESPGHAN guidelines recommending low-volume preparations [7], highlighting a gap between recommended standards and current clinical practice. Limited availability of preferred formulations, regulatory constraints, organizational factors, and the persistence of adult-derived protocols may explain this discrepancy. The coexistence of personalized approaches and partial guidelines adherence may therefore create uncertainty in selecting the optimal regimen for pediatric patients in Italy.

In this context, achieving an appropriate balance between personalization and evidence-based standardization appears essential. Future studies comparing different agents and strategies in pediatric populations, together with the development of national guidelines tailored to the Italian healthcare system, could support practice harmonization and ensure more consistent, evidence-aligned clinical decision-making.

A considerable proportion of centers also incorporate additional measures into the bowel preparation process. Osmotic laxatives were prescribed in 29 of the 39 centers, mainly to manage constipation or following a previous inadequate cleansing, although routine use was uncommon. Moreover, 69% of centers recommended a low-fiber diet in the days preceding the procedure, and rectal enemas were administered in cases of insufficient cleansing in 27 centers. These practices, however, are not fully in line with current ESGE/ESPGHAN recommendations, which do not support the routine use of additional osmotic laxatives or enemas before colonoscopy. Conversely, a low-residue diet is recommended as part of the standard preparation regimen [6].

The majority of colonoscopies were performed in the morning. Afternoon procedures adopted split-dose or modified preparation regimens. While multiple studies have demonstrated that split-dose preparations in children are more effective and better tolerated than day-before regimens, the issue of timing relative to pediatric sedation protocols has not been adequately addressed [10].

Accurate and standardized assessment of bowel cleansing is crucial for ensuring colonoscopy quality [7, 11]. Validated scoring systems, such as the BBPS, allow objective and reproducible evaluation, replacing subjective terminology such as “excellent,” “fair,” or “poor.” Although all centers reported the quality of bowel preparation in their endoscopy documentation, the adoption of validated scoring tools was not yet universal, indicating an area for improvement.

Effective patient and caregiver education plays a crucial role in achieving adequate bowel preparation for pediatric colonoscopy. Beyond traditional written and verbal instructions, current evidence supports the use of structured, multimodal, and age-appropriate educational strategies — including visual tools, digital resources, and interactive approaches — to enhance understanding, adherence, and ultimately the quality of bowel cleansing.

In line with this, ESGE recommendations encourage the adoption of engaging solutions such as visual aids, mobile applications, and reminder systems to improve preparation outcomes [2, 6].

We did not collect data on colonoscopy cancellation rates due to poor preparation. Missing this metric is an important limitation, as inadequate preparation can lead to prolonged procedures or cancellations, affecting resource use. Future studies should record cancellation rates to better assess the impact of preparation variability.

Overall, the survey highlights the need for national pediatric guidelines in Italy to standardize bowel preparation practices, optimize patient safety and comfort, and improve procedural outcomes.

This study provides a comprehensive overview of current bowel preparation practices across Italian pediatric centers. The inclusion of both medical and surgical units strengthens the generalizability of the findings. However, the voluntary nature of survey participation may introduce selection bias. Furthermore, the absence of patient-reported outcomes and objective measures of bowel cleansing effectiveness (e.g., BBPS scores distribution) limits clinical extrapolation.

Future steps should include multicenter trials comparing regimens, and the development of educational materials (e.g., leaflets, videos, and apps) to improve family engagement and adherence.

## Conclusions

This nationwide survey reveals substantial variability in bowel preparation practices across Italian pediatric centers, with overall suboptimal adherence to European guidelines. Although adverse events were infrequently reported, issues related to palatability, volume, and overall tolerability remain common. The development and implementation of standardized national guidelines could support the harmonization of clinical practice and strengthen future research, enabling a more accurate evaluation of the efficacy and safety of different preparations and administration strategies. Ultimately, improving patient adherence, enhancing colonoscopy quality, and integrating individualized, patient-centered approaches should represent key objectives for optimizing bowel preparation in children.

## Supplementary Information

Below is the link to the electronic supplementary material.

Supplementary Material 1

## Data Availability

Data are available from the corresponding author upon reasonable request.

## Abbreviations

BBPS: Boston bowel preparation scale
ESGE: European society for gastrointestinal endoscopy
ESPGHAN: European society for paediatric gastroenterology, hepatology and nutrition
SIGENP: Italian society of pediatric gastroenterology, hepatology and nutrition
PEG: Polyethylene glycol
PEG-ELS: Polyethylene glycol with electrolytes
