# Supplementary material for: Bowel preparation for colonoscopy in children: a survey of the Italian society of pediatric gastroenterology, hepatology and nutrition
Source: Ital J Pediatr. 2026 Mar 19;52:67. doi: 10.1186/s13052-026-02227-4 (PMC13122899; doi:10.1186/s13052-026-02227-4)
Supplement: Supplementary file 1 — Supplementary Material 1 [file 13052_2026_2227_MOESM1_ESM.docx]

**Supplementary material**

**Supplementary Table 1. Questionnaire on bowel preparation for colonoscopy in children**

| \| Question \| Responses \| \| --- \| --- \| \| What department do you work in? \| Pediatrics; Pediatric gastroenterology; Pediatric surgery; Other \| \| Who in your center performs the endoscopic examination? \| Adult gastroenterologist; Pediatric gastroenterologist; Pediatric surgeon \| \| Where are the procedures performed? \| Dedicated spaces; Surgical rooms \| \| How many colonoscopies are performed in your center per month? \| < 5; 5–10; 10–20; > 20 \| \| Where is the intestinal preparation performed? \| Always in the hospital; Always at home; Depends on the patient's characteristics \| \| What criteria do you use to decide whether the preparation should be performed at home or in the hospital? (e.g., age, comorbidities, compliance, other) \| Open answer \| \| Which preparation do you use for intestinal preparation? (Multiple answers are possible) \| Sodium Picosulfate (e.g., Picoprep, Citrafleet); Polyethylene glycol with electrolytes – high-volume (e.g., Selg Esse, Colirei, Isocolan); Polyethylene glycol with electrolytes – low-volume (e.g., Clensia, Plenvu, Moviprep); Polyethylene glycol without electrolytes \| \| Do you use any recommendations for bowel preparation in children (e.g., ESGE, ESPGHAN, NASPGHAN)? \| Yes; No \| \| If you use recommendations, please specify which ones. \| Open answer \| \| Do you use the preparation provided by your hospital? \| Yes; Yes, but I am not satisfied; No; Only in some cases \| \| Please specify when you replace the preparation provided by your hospital. \| Poor palatability; Volumes not suitable for age; Preparation not recommended for pediatric age; Previous ineffective use \| \| Specify why you are not satisfied with the preparation provided by the hospital. \| Open answer \| \| High-volume preparations based on macrogol and electrolytes (e.g., Selg Esse, Colirei, Isocolan) are contraindicated in children aged <8 years and weighing <20 kg. How do you behave? \| I use them in children of any age and weight; I only use them in children weighing >20 kg and aged >8 years; I don't use them \| \| Low-volume preparations based on macrogol and electrolytes (e.g., Plenvu, Clensia, Moviprep) are contraindicated in children under 18 years of age. How do you behave? \| I use them regularly; I don't use them \| \| Sodium Picosulfate (e.g., Citrafleet, Picoprep) is contraindicated in patients suffering from an active inflammatory bowel disease such as Crohn's disease or ulcerative colitis. How do you behave? \| I use it regardless of known or suspected intestinal pathology; I do not use it in patients with active IBD; I don't use it \| \| What methods do you use to provide information for bowel preparation? (Multiple answers are possible) \| Verbal instructions during the visit; Written instructions; Other \| \| Do you prescribe macrogol in the days before the preparation? (Multiple answers are possible) \| Yes, always; No, never; Only if the patient is constipated; Only if previous preparation was insufficient \| \| Do you prescribe a low-fiber diet (e.g., excluding vegetables, legumes, or fruit) in the days leading up to the preparation? \| Yes; No; Only in selected cases \| \| Do you perform a rectal enema before colonoscopy? \| Yes, always; No, never; Only if you believe the preparation may be insufficient \| \| Within what time frame do you require the patient to complete the intestinal preparation? \| Within 4–6 hours; No specific time frame; According to the instructions provided in the leaflet of the preparation used \| \| How long should the patient fast before the procedure? \| 4 hours for all liquids and 6 hours for solids; 1 hour for clear liquids, 4 hours for breast milk, 6 hours for cow’s milk, 6 hours for solids; Other \| \| At your center, colonoscopies are usually performed: \| In the morning; In the afternoon; In the morning and in the afternoon; Depending on the availability of the endoscopy or operating room \| \| What bowel preparation method do you use for colonoscopies scheduled in the afternoon? \| Split-dose preparation; Same-day preparation; Postpone the start of preparation as much as possible; Increase the volume of the standard preparation; Use an evacuative enema; Do not modify the bowel preparation regimen \| \| Do you use the nasogastric tube: \| Always in children under 5 years of age; Always in children under 10 years of age; Only if the child is unable to take the preparation orally \| \| In your experience, what are the most common issues caused by bowel preparation? (Multiple answers are possible) \| Nausea and vomiting; Refusal due to unpleasant taste; Difficulty in consuming large volumes; Abdominal cramps; All of the above \| \| Have you ever encountered adverse events during preparation, such as electrolyte disturbances, hypotension, or others? \| Yes; No \| \| Do you administer an antiemetic drug if nausea or vomiting occurs? \| Yes; No \| \| Which medication do you use in case of vomiting or nausea? \| Ondansetron; Metoclopramide; Domperidone; Chlorphenamine \| \| How do you describe bowel preparation in the endoscopic report? \| Using adjectives (adequate, inadequate, insufficient...); Using the Boston Scale; I do not describe it in the endoscopic report \| \| In your experience, is bowel preparation a stressful procedure for children? \| Yes; No \| \| Do you feel the need for Italian guidelines? \| Yes; No, I am satisfied with the protocol I currently use \| |  |
| --- | --- | --- | --- | --- | --- | --- | --- | --- | --- | --- | --- | --- | --- | --- | --- | --- | --- | --- | --- | --- | --- | --- | --- | --- | --- | --- | --- | --- | --- | --- | --- | --- | --- | --- | --- | --- | --- | --- | --- | --- | --- | --- | --- | --- | --- | --- | --- | --- | --- | --- | --- | --- | --- | --- | --- | --- | --- | --- | --- | --- | --- | --- | --- | --- | --- |
|  |  |

**Supplementary Table 2. - Geographical distribution of the participating centers (n= 39)**

| **Geographic Area** | **No. of centers (%)** | **Cities (no. of centers)** |
| --- | --- | --- |
| Northern Italy | 22 (56.4 %) | Alessandria (3), Bergamo, Como, Genova (2), Milano (3), Monza, Padova, Ravenna, Reggio Emilia, Rimini, Torino (2), Trento, Trieste, Varese, Verona, Vicenza |
| Central Italy | 9 (23.1 %) | Cesena, Chieti, Firenze, L’Aquila, Perugia, Roma (4) |
| Southern Italy and Islands | 8 (20.5 %) | Agrigento, Catania, Cosenza, Napoli (3), Palermo (2), |
